# Predominant patterns of β-lactam hypersensitivity in a single German Allergy Center: exanthem induced by aminopenicillins, anaphylaxis by cephalosporins

**DOI:** 10.1186/s13223-020-00488-0

**Published:** 2020-11-17

**Authors:** Philipp Schrüfer, Knut Brockow, Johanna Stoevesandt, Axel Trautmann

**Affiliations:** 1 Department of Dermatology and Allergy, Allergy Center Mainfranken, University Hospital Würzburg, 97080 Würzburg, Germany; 2 Department of Dermatology and Allergy, Technical University of Munich, 80802 Munich, Germany

**Keywords:** Amoxicillin, Ampicillin, Angioedema, Drug adverse reaction, Drug allergy, Drug hypersensitivity, Penicillin allergy, Penicillin hypersensitivity, Urticaria

## Abstract

**Background:**

Penicillins and other β-lactam antibiotics are the most common elicitors of allergic drug reaction. However, data on the pattern of clinical reaction types elicited by specific β-lactams are scarce and inconsistent. We aimed to determine patterns of β-latam allergy, i.e. the association of a clinical reaction type with a specific β-lactam antibiotic.

**Methods:**

We retrospectively evaluated data from 800 consecutive patients with suspected β-lactam hypersensitivity over a period of 11 years in a single German Allergy Center.

**Results:**

β-lactam hypersensitivity was definitely excluded in 595 patients, immediate-type (presumably IgE-mediated) hypersensitivity was diagnosed in 70 and delayed-type hypersensitivity in 135 cases. Most (59 out of 70, 84.3%) immediate-type anaphylactic reactions were induced by a limited number of cephalosporins. Delayed reactions were regularly caused by an aminopenicillin (127 out of 135, 94.1%) and usually manifested as a measles-like exanthem (117 out of 135, 86.7%). Intradermal testing proved to be the most useful method for diagnosing β-lactam allergy, but prick testing was already positive in 24 out of 70 patients with immediate-type hypersensitivity (34.3%). Patch testing in addition to intradermal testing did not provide additional information for the diagnosis of delayed-type hypersensitivity. Almost all β-lactam allergic patients tolerated at least one, usually several alternative substances out of the β-lactam group.

**Conclusions:**

We identified two patterns of β-lactam hypersensitivity: aminopenicillin-induced exanthem and anaphylaxis triggered by certain cephalosporins. Intradermal skin testing was the most useful method to detect both IgE-mediated and delayed-type β-lactam hypersensitivity.

## Background

Among commonly used drugs, β-lactam antibiotics, particularly penicillins and cephalosporins, are most frequently associated with an allergic hypersensitivity reaction [1]. Up to 10% of the population claim to be allergic to penicillin [2] though penicillin allergy can be confirmed in only a minority of cases. The negative impact of unverified penicillin allergy on future antibiotic treatment and subsequent health care costs was clearly demonstrated [3, 4].

The sheer number of cases impedes thorough diagnostic work-up of all patients claiming to be allergic to β-lactam antibiotics by an allergy specialist. Once an acute infection requires immediate antibiotic treatment, there is usually no time for testing, and/or equipment and specialist knowledge are not available. The clinical picture and diagnosis of an allergic reaction induced by penicillin or other β-lactam antibiotics is comprehensively presented in reviews and guidelines [5, 6]. However, it remains unknown whether all individual β-lactams are regularly associated with the entire clinical spectrum of drug hypersensitivity reactions.

With the present data evaluation in a single Allergy Center, we aimed: (i) To identify clinical reaction patterns and assess their association with specific β-lactam antibiotics. (ii) To assess the utility of currently available diagnostic methods for different reaction types. (iii) To evaluate whether patients with confirmed penicillin or cephalosporin allergy tolerate an alternative β-lactam.

## Methods

### Patients

We retrospectively evaluated 800 patients referred to our Allergy Center from January 2009 to December 2019 for diagnostic work-up of an immediate-type or delayed-type hypersensitivity reaction attributed to a β-lactam antibiotic by the treating physician. The severity of immediate reactions (i.e. anaphylaxis) was classified as mild, moderate or severe as specified in Additional file 1 [7]. Delayed reactions included measles-like (maculo-papular) exanthem, symmetrical drug-related intertriginous and flexural exanthem (SDRIFE), fixed drug eruption (FDE), and drug reaction with eosinophilia and systemic symptoms (DRESS). The institutional review board of the University Hospital Würzburg consented to the retrospective review and publication of anonymized data.

### β-lactam-specific serum IgE

Immunoglobulin E binding to benzyl penicilloyl, phenoxymethyl penicilloyl, amoxicilloyl, ampicilloyl, and cefaclor was determined by ImmunoCAP (Thermo Fisher Scientific, Freiburg, Germany) [8]. A value of > 0.35 kU_A_/L was considered significant only in patients with a convincing history of an IgE-mediated reaction, i.e. the anaphylaxis spectrum.

### β-lactam skin testing

Skin testing was performed according to international guidelines and included reading at 15 min in suspected immediate reactions and additional readings on days two, three, and four in delayed reactions [9]. Patients were tested with a series of different penicillins and cephalosporins; the concentrations used for patch, prick, and intradermal skin testing are detailed in Additional file 2. Test concentrations for intradermal testing are within the range recommended in guidelines, for prick and patch testing comparatively high concentrations were used in our Allergy Center (Additional file 2). At the 15 min reading, a wheal of more than 3 mm in diameter with surrounding erythema was considered a positive prick test result, and a wheal of at least 6 mm was assessed as positive in intradermal testing. An erythematous and infiltrated plaque or eczematous lesion, clearly visible and palpable on days two, three, and four was interpreted as a positive delayed-type skin test reaction [10].

### β-lactam challenge testing

Patients with negative β-lactam-specific IgE and negative skin test results underwent diagnostic challenge. Challenge testing was also performed to identify an alternative tolerated β-lactam antibiotic in patients with proven β-lactam allergy. General principles of our protocol are based on guideline recommendations [11]: (i) Provocation testing was performed after a minimum of 6 weeks following a delayed reaction, and a minimum of 2 weeks following an immediate reaction. (ii) β-lactam doses were incrementally increased to an average daily dose and adapted to age, kidney function or weight if necessary (Additional file 2). (iii) Intervals of 30 min were kept between individual doses. (iv) Patients were observed for at least four hours after the last dose and were advised to present for objective examination if any symptoms developed within the next hours or days. Primary aim of the prolonged monitoring was not only to observe a reaction (IgE-mediated anaphylaxis will develop within 30 min, a delayed-type reaction at earliest after several hours) but rather to calm down the oftentimes quite anxious patient before he leaves definitely the Allergy Center.

## Results

In 205 out of total 800 evaluated patients, a diagnosis of β-lactam hypersensitivity could be established in our Allergy Center, based on an overall assessment including history, the clinical reaction and results of testing. β-lactam hypersensitivity could be definitely excluded in the remaining 595 cases by negative serum IgE and negative skin testing followed by challenge of the incriminated β-lactam antibiotic (data not shown). The time interval between the β-lactam associated reaction and allergy testing of all 800 patients was ≤ 1 year in 491 cases, > 1 to 5 years in 79, > 5 to 10 years in 30, > 10 years in 181, unclear or not sufficiently documented in 19.

### Immediate-type β-lactam hypersensitivity

**Fig. 1:**
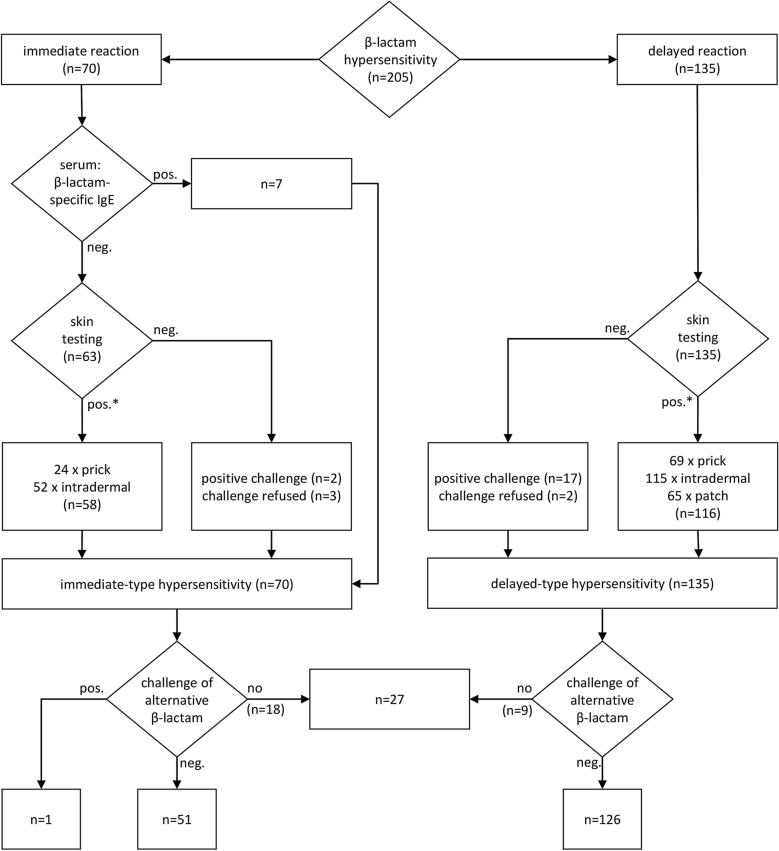
Diagnostic work-up of 205 patients with β-lactam hypersensitivity. * More than one positive skin test result per case possible, e.g. positive prick as well as positive intradermal test (see also Table 2)

Out of 205 cases with β-lactam hypersensitivity, an immediate-type (presumably IgE-mediated) reaction was diagnosed in 70 (34.1%) (Table 1, Fig. 1). Diagnosis was based on positive testing (i.e. prick testing, intradermal testing, serum IgE) together with a convincing history in 65 cases (92.9%), in two on a positive challenge and in the remaining three on history alone. Twenty-six patients developed predominantly urticaria/angioedema with negligible to minor systemic signs; 44 moderate to severe anaphylaxis. Fifty-nine out of the 70 immediate reactions were induced by a cephalosporin (84.3%) (Table 1). In 34 cases with immediate-type β-lactam hypersensitivity, the β-lactam antibiotic was administered intravenously; 24 out of these developed an intraoperative anaphylactic incident during general anesthesia.

**Table 1:** Diagnosis, causal as well as tolerated β-lactam antibiotic in 205 patients with β-lactam hypersensitivity

	Immediate-type hypersensitivity (n = 70)	Delayed-type hypersensitivity (n = 135)
Males/females	28 / 42	48 / 87
Median age (range, years)	47 (11–76)	50 (7–83]
Anaphylaxis		
Mild	26	n.a.
Moderate	27	
Severe	17	
Delayed reaction		
Measles-like exanthem	n.a.	117
SDRIFE		12
FDE		3
DRESS		3
Causal β-lactam antibiotic		
Aminopenicillin (amoxicillin or ampicillin)	3	91
Aminopenicillin + benzyl penicillin	2	36
Cefuroxime	12	2
Cefazolin	9	0
Ceftriaxone	4	1
Cefaclor	4	0
Several cephalosporins	18	1
Cephalosporin + aminopenicillin	12	0
Benzyl / phenoxymethyl penicillin	3	3
Piperacillin + tazobactam	3	0
Flucloxacillin	0	1
Tolerated β-lactam antibiotics (several different β-lactams per case possible)		
Aminopenicillin (amoxicillin or ampicillin)	31	4
Cephalosporin	58	179
Phenoxymethyl penicillin	32	48

DRESS, drug reaction with eosinophilia and systemic symptoms; FDE, fixed drug eruption; SDRIFE, symmetrical drug-related intertriginous and flexural exanthem; n.a., not applicable

### Delayed-type β-lactam hypersensitivity

Out of 205 cases, delayed-type β-lactam hypersensitivity was diagnosed in 135 patients (65.9%). Out of these, 117 developed a measles-like exanthem (86.7%) and 12 a SDRIFE reaction pattern (8.9%) (Table 1). A comparatively small number of cases were diagnosed as FDE (n = 3) or DRESS (n = 3). Diagnosis of these forms of a drug hypersensitivity reaction was based on established clinical and laboratory criteria [12–14]. The three DRESS cases developed a skin eruption compatible with acute generalized exanthematous pustulosis (AGEP), but due to accompanying hepatitis the reaction had to be classified finally as DRESS [13]. Most delayed reactions (n = 127, 94.1%) were caused by the aminopenicillins amoxicillin or ampicillin. Thirty-six out of the 127 patients (28.3%) with delayed-type aminopenicillin hypersensitivity were concomitantly sensitized to benzyl penicillin (Table 1).

### β-lactam-specific serum IgE and skin testing

Test results are depicted in detail in Table 2 and are summarized in Fig. 1. In 24 out of 70 cases (34.3%) with immediate-type β-lactam hypersensitivity, the prick test was clearly positive after 15 min. Thirty-four (48.6%) cases of immediate-type β-lactam hypersensitivity could only be detected by intradermal testing, the prick test yielded a (false) negative result (Table 2). In seven cases, the diagnosis of IgE-mediated allergy was based on the detection of β-lactam-specific serum IgE together with a convincing history (Table 2, Fig. 1). One-hundred-fifteen out of 135 patients (85.2%) with delayed-type hypersensitivity had a positive intradermal test. Skin prick testing was also positive in 68 of 115 intradermal test-positive individuals (59.1%) with delayed-type hypersensitivity.

**Table 2:** Results of β-lactam-specific serum IgE, skin and challenge testing

	Serum IgE	Prick	Intradermal	Patch	Challenge	Cases (n)
Immediate-type hypersensitivity (n = 70)	** + **	n.d	n.d	n.a	n.d	7
	–	** + **	n.d		n.d	6
	–	** + **	** + **		n.d	18
	–	–	** + **		n.d	34
	–	–	–		** + **	2
	–	–	–		Refused	3
Delayed-type hypersensitivity (n = 135)	n.a.	** + **	** + **	–	n.d	6
		** + **	** + **	n.d	n.d	8
		** + **	** + **	** + **	n.d	54
		** + **	n.d	–	n.d	1
		–	** + **	–	n.d	33
		–	** + **	** + **	n.d	11
		–	** + **	n.d	n.d	3
		–	–	–	** + **	17
		–	–	–	Refused	2

+ , Positive result; –, negative result; n.a., not applicable; n.d., not done. The criteria defining positive serum IgE, positive prick, positive intradermal, and positive patch testing are described in the Sect ."Methods"

### Diagnostic β-lactam challenge testing

Both, prick and intradermal testing were false negative in only 2 out of 70 cases of immediate-type β-lactam hypersensitivity (2.9%), and the diagnosis was confirmed by challenge which triggered an anaphylactic reaction (Table 2, Fig. 1). In 17 skin test-negative patients, delayed-type hypersensitivity was confirmed by positive challenge; thirteen patients developed measles-like exanthem, three a SDRIFE, and one FDE.

### Challenge testing to identify an alternative tolerated β-lactam antibiotic

In 177 out of 205 cases with β-lactam hypersensitivity (86.3%), we identified at least one tolerated alternative β-lactam antibiotic by challenge testing (Fig. 1). Patients with delayed-type aminopenicillin hypersensitivity tolerated at least one, mostly two or three alternative cephalosporins without an aminobenzyl R1 side chain, i.e. cefuroxime, cefazolin, and ceftriaxone (Table 1). In patients with immediate-type cephalosporin hypersensitivity, challenge testing proved regularly tolerance of another cephalosporin carrying a different R1 side chain, phenoxymethyl penicillin, and aminopenicillins (Table 1).

## Discussion

Our data from a single German Allergy Center indicate that immediate anaphylactic reactions were usually triggered by a cephalosporin, whereas the aminopenicillins amoxicillin and ampicillin predominantly caused an exanthematous skin rash. Importantly, for all reaction types and β-lactams, intradermal testing seems to be the most useful method to detect sensitization.

Single cephalosporins including cefuroxime, cefazolin, and ceftriaxone were responsible for the majority of (presumably IgE-mediated) immediate-type hypersensitivity reactions to β-lactam antibiotics in our series. Single shot intravenous administration of cephalosporins has accordingly been identified as an important elicitor of intraoperative anaphylaxis leading to hypotension and increased ventilation pressure [15, 16].

The different clinical forms of delayed-type β-lactam hypersensitivity are comprehensively described and discussed in a number of recent reviews [5, 6, 17]. Surprisingly, the by far most common clinical manifestation, measles-like exanthem, receives relatively little attention [18]. The term uncomplicated exanthem has been introduced in order to underline the absence of severe systemic involvement. Sub-febrile temperature or a slight increase of liver enzymes are occasionally observed during episodes of an exanthematous rash, but may also be caused by the infectious disease treated with the β-lactam antibiotic. Aminopenicillin-induced measles-like exanthem does not belong to the spectrum of severe drug reactions, and the fear that Stevens-Johnson syndrome or toxic epidermal necrolysis will develop, if the causative aminopenicillin is continued or re-administered is not justified [19].

According to international standards, we used a commercial immunoassay to determine allergen-specific IgE [8, 20]. Cefaclor is currently the only available cephalosporin for ImmunoCAP testing. Our data, however, show, that immediate-type β-lactam hypersensitivity is mainly triggered by other cephalosporins including ceftriaxone, cefazolin or cefuroxime, for which no validated serological IgE test exits [21]. Additional laboratory methods including the basophil activation test or the lymphocyte transformation test may yield sensitive and specific results, e.g. in the diagnosis of aminopenicillin-induced exanthem [22, 23].

The most powerful method to detect β-lactam allergy in our data evaluation was intradermal testing. As a requirement, the respective β-lactam must be available for intravenous administration, and 1:10 or a higher dilution is needed to obtain a non-irritating test concentration. The intradermal test concentrations used in our series are within the range of published standards [9]. Prick testing can be done with undiluted β-lactams including tablets but is commonly considered to be diagnostically less helpful than intradermal testing [24]. Our current data, however, show that a reasonable proportion of patients with immediate-type β-lactam hypersensitivity (34.3%) has a positive prick test result. It was postulated that skin testing for the diagnosis of delayed (non-immediate) reactions to β-lactam antibiotics may be optimized by a combination of prick, intradermal, and patch tests [17]. Patch testing in our series was only positive in patients with a concurrently positive intradermal test and, therefore, did not add to the overall utility of testing and might be dispensable.

According to current recommendations, oral β-lactam antibiotics were preferably used for challenge testing in our series if available. Intravenous challenge, however, has the clear advantage that the infusion can be stopped immediately in case of an anaphylactic reaction [11]. The optimal dose for challenge testing is still controversially debated. A certain, albeit low threshold dose, is required in order to reliably elicit an objective clinical reaction in allergic individuals. Our protocol includes the incremental increase to an average daily dose of the respective β-lactam, which should be sufficient to trigger symptoms in a sensitized patient. The risk of sensitization or an immunological boost by challenge testing is estimated to be very low [25, 26]. Accordingly, we are not aware of any patient that reacted again to a β-lactam antibiotic which was previously tolerated in our challenge procedure.

Co-sensitization to numerous or even all different β-lactam antibiotics seems to be extremely rare. The side chain structure of the β-lactam ring of penicillins (R at C6) and cephalosporins (R1 at C7) seems to be the most important antigenic determinant [27]. The structural similarity of the R side chain of aminopenicillins and benzyl penicillin probably explains the 28.3% rate of cross-reactions observed in this series. The cross-reactivity between aminopenicillins and certain (amino) cephalosporins, namely cefalexin, cefaclor, and cefadroxil, may be attributed to an identical or very similar R and R1 side chain [28]. In this series and in accordance with published data, we demonstrate that patients with aminopenicillin allergy almost always tolerate cephalosporins with a different R1 side chain including cefazolin, ceftriaxone, and cefuroxime [29, 30].

### Limitations of our study

Data were retrospectively extracted from patient records, resulting in a certain methodological inhomogeneity, e.g. not all patients were examined with all skin test methods. The frequency of certain β-lactam antibiotics as a trigger of an allergic reaction depends on general usage and prescription rate. Consequently, our data from a single German Allergy Center may not be generalizable to other regions or study centers.

## Conclusions

i. In this patient series, immediate-type β-lactam hypersensitivity was most commonly triggered by single cephalosporins including cefuroxime, cefazolin, and ceftriaxone, frequently as full-blown anaphylaxis.
ii. The aminopenicillins amoxicillin and ampicillin were leading elicitors of delayed-type β-lactam hypersensitivity. They mainly caused measles-like exanthem; in contrast to other series, other forms of a delayed drug reaction including SDRIFE, FDE, and DRESS were far less common.
iii. Intradermal testing of β-lactam antibiotics in a non-irritating dilution was the most powerful method for the diagnosis of both immediate and delayed-type β-lactam hypersensitivity. Preliminary prick testing detected IgE-mediated allergy in almost one third of cases. In our hands, additional patch testing does not add to the overall utility of skin testing.
iv. Almost all our patients with proven β-lactam allergy tolerated at least one, usually several alternative substances out of the β-lactam group.

## Supplementary information

**Additional file 1.** Grading the severity of anaphylaxis [modified from (7)].

**Additional file 2.** Concentration of β-lactam antibiotics for skin testing and sequence of increasing doses used in single-blinded challenge. * For prick and patch testing, tablets were ground in a mortar and suspended with 1 mL physiological saline solution. iv, intravenous; n.d., not done.

## Data Availability

Not applicable (this is a retrospective data evaluation).

## Abbreviations

DRESS: Drug reaction with eosinophilia and systemic symptoms
FDE: Fixed drug eruption
SDRIFE: Symmetrical drug-related intertriginous and flexural exanthem

## References

[1] Demoly P, Adkinson NF, Brockow K (2014). International consensus on drug allergy. Allergy.

[2] Macy E (2014). Penicillin and beta-lactam allergy: epidemiology and diagnosis. Curr Allergy Asthma Rep.

[3] Li M, Krishna MT, Razaq S, Pillay D (2014). A real-time prospective evaluation of clinical pharmaco-economic impact of diagnostic label of 'penicillin allergy' in a UK teaching hospital. J Clin Pathol.

[4] Macy E, Contreras R (2014). Health care use and serious infection prevalence associated with penicillin "allergy" in hospitalized patients: A cohort study. J Allergy Clin Immunol.

[5] Mirakian R, Leech SC, Krishna MT (2015). Management of allergy to penicillins and other beta-lactams. Clin Exp Allergy.

[6] Blanca M, Romano A, Torres MJ (2009). Update on the evaluation of hypersensitivity reactions to betalactams. Allergy.

[7] Brown SG (2004). Clinical features and severity grading of anaphylaxis. J Allergy Clin Immunol.

[8] Blanca M, Mayorga C, Torres MJ (2001). Clinical evaluation of Pharmacia CAP System RAST FEIA amoxicilloyl and benzylpenicilloyl in patients with penicillin allergy. Allergy.

[9] Brockow K, Garvey LH, Aberer W (2013). Skin test concentrations for systemically administered drugs – an ENDA/EAACI Drug Allergy Interest Group position paper. Allergy.

[10] Friedmann PS, Ardern-Jones M (2010). Patch testing in drug allergy. Curr Opin Allergy Clin Immunol.

[11] Aberer W, Bircher A, Romano A (2003). Drug provocation testing in the diagnosis of drug hypersensitivity reactions: general considerations. Allergy.

[12] Flowers H, Brodell R, Brents M, Wyatt JP (2014). Fixed drug eruptions: presentation, diagnosis, and management. South Med J.

[13] Kano Y, Shiohara T (2009). The variable clinical picture of drug-induced hypersensitivity syndrome/drug rash with eosinophilia and systemic symptoms in relation to the eliciting drug. Immunol Allergy Clin North Am.

[14] Häusermann P, Harr T, Bircher AJ (2004). Baboon syndrome resulting from systemic drugs: is there strife between SDRIFE and allergic contact dermatitis syndrome?. Contact Dermatitis.

[15] Volcheck GW, Hepner DL (2019). Identification and management of perioperative anaphylaxis. J Allergy Clin Immunol Pract.

[16] Trautmann A, Seidl C, Stoevesandt J, Seitz CS (2016). General anaesthesia-induced anaphylaxis: impact of allergy testing on subsequent anaesthesia. Clin Exp Allergy.

[17] Romano A, Blanca M, Torres MJ (2004). Diagnosis of nonimmediate reactions to beta-lactam antibiotics. Allergy.

[18] Brockow K, Ardern-Jones MR, Mockenhaupt M (2019). EAACI position paper on how to classify cutaneous manifestations of drug hypersensitivity. Allergy.

[19] Trautmann A, Benoit S, Goebeler M, Stoevesandt J (2017). "Treating through" Decision and follow-up in antibiotic therapy-associated exanthemas. J Allergy Clin Immunol Pract.

[20] Macy E, Goldberg B, Poon KY (2010). Use of commercial anti-penicillin IgE fluorometric enzyme immunoassays to diagnose penicillin allergy. Ann Allergy Asthma Immunol.

[21] Uyttebroek AP, Decuyper II, Bridts CH (2016). Cefazolin hypersensitivity: toward optimized diagnosis. J Allergy Clin Immunol Pract.

[22] Mayorga C, Celik G, Rouzaire P (2016). In vitro tests for drug hypersensitivity reactions: an ENDA/EAACI Drug Allergy Interest Group position paper. Allergy.

[23] Trautmann A, Seitz CS, Stoevesandt J, Kerstan A (2014). Aminopenicillin-associated exanthem: lymphocyte transformation testing revisited. Clin Exp Allergy.

[24] Torres MJ, Romano A, Mayorga C (2001). Diagnostic evaluation of a large group of patients with immediate allergy to penicillins: the role of skin testing. Allergy.

[25] Solensky R, Earl HS, Gruchalla RS (2002). Lack of penicillin resensitization in patients with a history of penicillin allergy after receiving repeated penicillin courses. Arch Intern Med.

[26] Ponvert C, Weilenmann C, Wassenberg J (2007). Allergy to betalactam antibiotics in children: a prospective follow-up study in retreated children after negative responses in skin and challenge tests. Allergy.

[27] Pichichero ME, Zagursky R (2014). Penicillin and cephalosporin allergy. Ann Allergy Asthma Immunol.

[28] Blanca M, Mayorga C, Torres MJ (2002). Side-chain-specific reactions to betalactams: 14 years later. Clin Exp Allergy.

[29] Trcka J, Seitz CS, Bröcker EB, Gross GE, Trautmann A (2007). Aminopenicillin-induced exanthema allows treatment with certain cephalosporins or phenoxymethyl penicillin. J Antimicro Chemother.

[30] Romano A, Valluzzi RL, Caruso C, Zaffiro A, Quaratino D, Gaeta F. Tolerability of cefazolin and ceftibuten in patients with IgE-mediated aminopenicillin allergy. J Allergy Clin Immunol Pract 2020. 10.1016/j.jaip.2020.02.025 32145403

