# Supplementary material for: Predominant patterns of β-lactam hypersensitivity in a single German Allergy Center: exanthem induced by aminopenicillins, anaphylaxis by cephalosporins
Source: Allergy Asthma Clin Immunol. 2020 Nov 17;16:102. doi: 10.1186/s13223-020-00488-0 (PMC7672956; doi:10.1186/s13223-020-00488-0)
Supplement: Supplementary file 1 — Additional file 1. Grading the severity of anaphylaxis [modified from (7)]. [file 13223_2020_488_MOESM1_ESM.doc]

# Additional file 1. Grading the severity of anaphylaxis [modified from (7)].

| **severity** | **symptoms** |
| --- | --- |
| **mild**: predominantly skin and subcutaneous tissue, minor systemic symptoms | urticaria with or without angioedema, throat tightness, tachycardia, mild abdominal discomfort |
| **moderate**: features of considerable respiratory, cardiovascular and/or gastrointestinal involvement | dysphonia or hoarseness, deep cough or wheezing, dyspnea, hypotension (systolic blood pressure <90 mmHg in adults), abdominal cramps, vomiting, somnolence, confusion, impaired vision |
| **severe**: hypoxia, shock, and severe neurological compromise | stridor, cyanosis, loss of sphincter control, syncope (loss of consciousness), respiratory and cardiac arrest |
