# Supplementary material for: Predominant patterns of β-lactam hypersensitivity in a single German Allergy Center: exanthem induced by aminopenicillins, anaphylaxis by cephalosporins
Source: Allergy Asthma Clin Immunol. 2020 Nov 17;16:102. doi: 10.1186/s13223-020-00488-0 (PMC7672956; doi:10.1186/s13223-020-00488-0)
Supplement: Supplementary file 2 — Additional file 2. Concentration of β-lactam antibiotics for skin testing and sequence of increasing doses used in single-blinded challenge. * For prick and patch testing, tablets were ground in a mortar and suspended with 1 mL physiological saline solution. iv, intravenous; n.d., not done. [file 13223_2020_488_MOESM2_ESM.doc]

# Additional file 2. Concentration of β-lactam antibiotics for skin testing and sequence of increasing doses used in single-blinded challenge. * For prick and patch testing, tablets were ground in a mortar and suspended with 1 mL physiological saline solution. iv, intravenous; n.d., not done.

| **skin testing** | **form** | **prick and patch [mg/mL]*** | **intradermal [mg/mL]; (dilution)** |
| --- | --- | --- | --- |
| benzyl penicillin | iv solution | 60 | 6 (1:10) |
| phenoxymethyl penicillin | tablet | 700 | n.d. |
| amoxicillin | tablet | 500 | n.d. |
| ampicillin | iv solution | 50 | 5 (1:10) |
| flucloxacillin | iv solution | 50 | 5 (1:10) |
| piperacillin + tazobactam | iv solution | 100 | 10 (1:10) |
| cefaclor | tablet | 500 | n.d. |
| cefuroxime | iv solution | 75 | 7,5 (1:10) |
| ceftriaxone | iv solution | 100 | 10 (1:10) |
| cefazolin | iv solution | 200 | 20 (1:10) |
| **challenge testing** | **administration** | **single doses [mg]** | **total dose [mg]** |
| phenoxymethyl penicillin | oral | 87.5; 175; 350; 700 | 1312.5 |
| amoxicillin | oral | 250; 500; 1000 | 1750 |
| ampicillin | oral | 250; 500; 1000; 2000 | 3750 |
| piperacillin + tazobactam | iv | 80; 200; 3720 | 4000 |
| cefaclor | oral | 62.5; 125; 250; 500 | 877.5 |
| cefuroxime | oral | 62.5; 125; 250 | 437.5 |
| ceftriaxone | iv | 20; 80; 100; 500; 1000 | 1700 |
| cefazolin | iv | 10; 40; 100; 1850 | 2000 |
